# Comparison of ILM peeling vs. inverted ILM flap for macular hole closure and visual outcomes: systematic review and meta-analysis

**DOI:** 10.1186/s40942-025-00707-z

**Published:** 2025-07-17

**Authors:** Reynaldo, Nadilla Garyudanefi, Puranto Budi Susetyo

**Affiliations:** 1 Ophthalmology Training Program, Gatot Soebroto Central Army Hospital, Mandala Tengah No. 25, Tomang, Jakarta, 11440 Indonesia; 2 Gatot Soebroto Central Army Hospital, Jakarta, Indonesia

**Keywords:** Macular hole, ILM peeling, Inverted ILM flap, Pars plana vitrectomy

## Abstract

**Background:**

A macular hole (MH) is a retinal condition affecting the central macula, leading to progressive visual impairment. Pars plana vitrectomy with internal limiting membrane (ILM) peeling is the standard surgical treatment, while the inverted ILM flap technique has emerged as a promising alternative. However, the effectiveness of this technique was still debated.

**Methods:**

Randomized controlled trials (RCTs) comparing ILM peeling and inverted ILM flap for MH were identified through searches in PubMed, ScienceDirect, Cochrane Library, and ClinicalTrials.gov in the last 15 years that compared ILM peeling and inverted ILM flap procedure. The primary outcome was anatomical closure, and the secondary outcome was visual acuity (VA) post-procedure. Data synthesis was performed using Review Manager (RevMan) 5.4.1 with odds ratio (OR) for anatomical closure and mean difference (MD) for VA with 95% confidence interval (CI). Statistical significance is achieved when the p-value is below 0.05.

**Results:**

Twelve RCTs involving 719 patients were included. The inverted ILM flap showed superior anatomical closure (OR 0.28; 95% CI: 0.15–0.52; *p* < 0.0001). VA post-procedure, based on follow-up time (3-, 6-, and 12-month), revealed no statistically significant difference in visual outcomes. Sensitivity analyses confirmed anatomical and visual benefits of the inverted flap in large MHs (≥ 400 μm).

**Conclusion:**

The inverted ILM flap technique offers better anatomical outcomes than ILM peeling, especially for larger MHs. Visual improvement is variable and may depend on MH chronicity and retinal recovery. Further high-quality studies are needed to confirm these findings.

**Supplementary Information:**

The online version contains supplementary material available at 10.1186/s40942-025-00707-z.

## Introduction

The macular hole (MH) is a retinal condition that affects the central foveal region and can potentially lead to vision loss or blindness. The incidence of MH is approximately 3.3 per 1.000 individuals, and MH is more common in older women, typically those over the age of 50. The exact pathophysiology of MHs remains unclear. Symptoms usually develop gradually and worsen over time, with patients often experiencing a decline in VA, metamorphopsia (visual distortion), and the appearance of a dark spot in the central field of vision [1].

There are two main types of MHs: traumatic MHs, resulting from blunt ocular trauma, and the more commonly encountered idiopathic MHs. Idiopathic MHs are caused primarily by vitreous traction at the center of the fovea in both the anteroposterior and the tangential directions [1, 2].

The management of MHs via pars plana vitrectomy with gas tamponade was first introduced by Kelly and Wendel in 1991 [3]. In 1997, the procedure evolved to internal limiting membrane (ILM) peeling to improve anatomical closure rates [4]. In 2010, Michalewska introduced the inverted ILM flap technique for managing MHs, which has since been associated with improved anatomical and visual outcomes [5]. In addition to this technique, several other innovative approaches have emerged in recent years, including autologous ILM transplantation, the lens capsular flap technique, neurosensory retinal grafts, amniotic membrane transplantation, retinotomy, and hydrodissection, offering alternative strategies for challenging and refractory MH cases [6].

This systematic review and meta-analysis aimed to evaluate the effectiveness of two different surgical approaches, ILM peeling and the inverted ILM flap, in treating MHs. The analysis focused on anatomical closure and functional outcomes measured by postoperative VA improvement. These findings are intended to provide further insight into selecting the best surgical technique in clinical practice.

## Materials and methods

### Study protocol

This study follows the Preferred Reporting Items for Systematic Review and Meta-Analyses (PRISMA) guidelines. The study protocol was submitted to PROSPERO, and the PROSPERO code is CRD420251022063. This study was conducted in five stages: (1) study design and search strategy; (2) assessment of the inclusion and exclusion criteria; (3) gathering of the articles; (4) Qualitative evaluation of the collected data; and (5) statistical analysis of the data.

### Search strategy

A comprehensive search of articles was conducted via PubMed, Science Direct, the Cochrane Library, and ClinicalTrials.gov up to April 2025 in the last 15 years. Articles were assessed based on the PICO framework: patients with macular holes (population), ILM peeling (intervention), Inverted ILM flap (comparison), and anatomical closure, or VA postprocedure (outcome). Searching was performed via keyword combinations relevant to the PICO components. All the authors screened all the articles by title and abstract via Rayyan, then screened the full texts to determine eligibility. All the citations were managed via Mendeley, and disagreements were resolved via discussion or consultation with a third author. The detailed search strategy is shown in the additional file [see additional file 1].

### Inclusion and exclusion criteria

The inclusion criteria were RCTs published in the last 15 years, patients with MHs, and articles that compared ILM peeling and inverted ILM flap procedures with anatomical closure and/or VA improvement outcomes. The exclusion criteria were articles that did not use a randomized controlled trial (RCT) design, such as systematic reviews, case reports, abstracts, or meta-analyses, and did not compare ILM peeling and inverted ILM flaps.

### Data extraction

Each article that meets the inclusion criteria undergoes a rigorous evaluation, and the required data will be compiled into one. The extracted data included the author, year of publication, study design, surgical technique, number of patients, average age, minimum MH diameter, anatomical closures, and best corrected visual acuity (BCVA) postprocedure. All twelve of these articles were RCTs.

### Quality assessment

Each author (R, N. G., and P. B. S.) assessed the quality of each article via the Cochrane risk of bias tool (RoB 2) [7]. If any differences of opinion were found, the authors discussed resolving the discrepancies in the results. A funnel plot was used to assess publication bias.

### Statistical analysis

Data evaluation for the meta-analysis using Review Manager 5.4.1. Continuous data, such as improvement in VA, is assessed using the mean difference (MD) with a 95% confidence interval (CI). Dichotomous data, namely anatomical closure, were assessed with the odds ratio (OR) with 95% CI. %. A random-effect model is used to analyze the results in the data with heterogeneity *P* < 0.05 and I^2^ > 50. Conversely, if *P* > 0.05 and I^2^ < 50%, then fixed-effect is used to analyze the data processing results [8, 9]. The data were converted into means and standard deviations via mean-variance estimation in articles that provide only median data with first and third quartiles [10, 11].

## Results

### Characteristics of the included studies

**Fig. 1:**
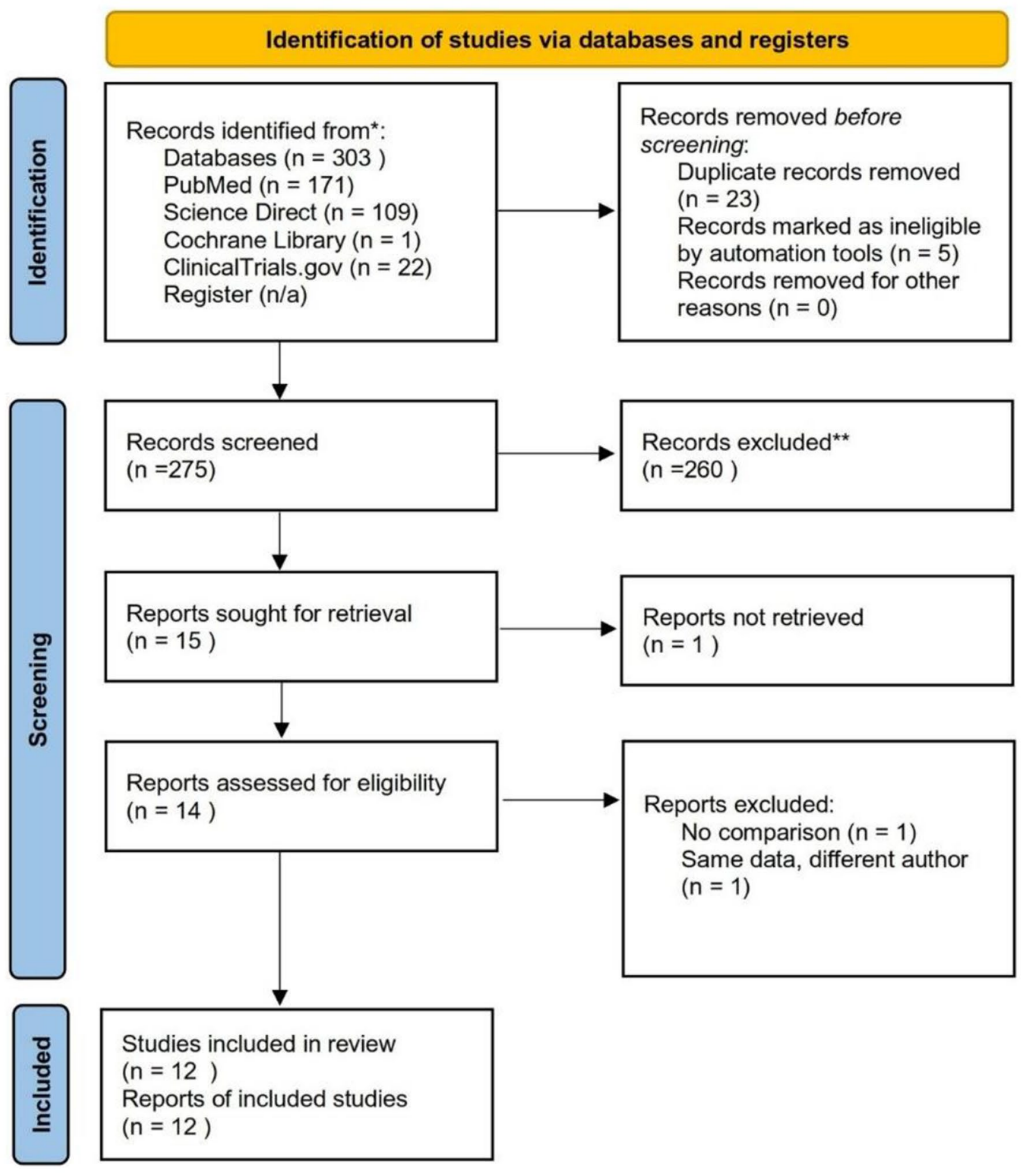
Article screening and retrieval following the PRISMA 2020 flowchart

Three hundred articles were successfully collected, and 12 studies met the inclusion criteria. A total of 709 patients were included, comprising 355 patients with ILM peeling and 354 patients with inverted ILM flaps. The screening and selection results of the PRISMA flow chart are shown in Fig. 1. The characteristics of the 12 RCTs can be seen in the additional file [see additional file Table 1]. Please see below for more information. One study reported results only at the 6th month [12], one study 1st and 6th months [13], three studies in the 1st and 3rd months [14–16], one study in the 1st, 3rd, 6th, and 12th months [17], two studies in the 12th month [5, 18], one study only in the 3rd month [19], one study in the 3rd, 6th, and 12th months [20], and one study in the 1st, 3rd, and 6th months [21].

### Quality assessment

**Fig. 2:**
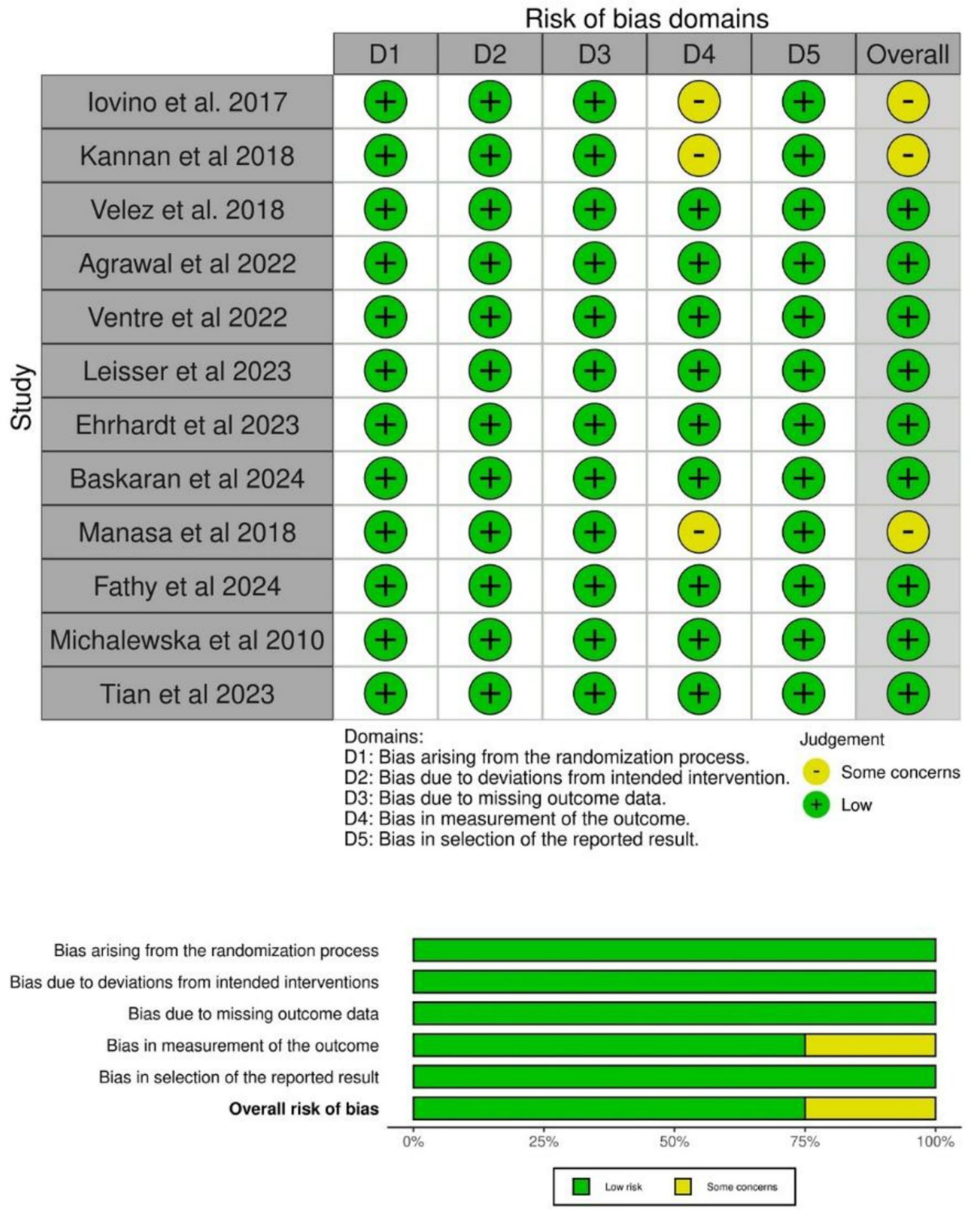
Summary risk of bias 12 included studies

Among the twelve studies included, the risk of bias assessment indicated that only three studies showed some concern [12, 13, 16], whereas the remaining studies were considered low risk. This study was retained from the systematic review and meta-analysis despite being assessed as having some concerns regarding the risk of bias, particularly in the domain of outcome measurement. Nevertheless, the study fulfilled all predefined inclusion criteria and contributed relevant data on anatomical closure and visual outcomes. Its inclusion ensures a more comprehensive synthesis of the existing evidence, with potential limitations appropriately addressed through risk of bias assessment and planned sensitivity analyses. See Fig. 2.

### Overall anatomical closure

**Fig. 3:**
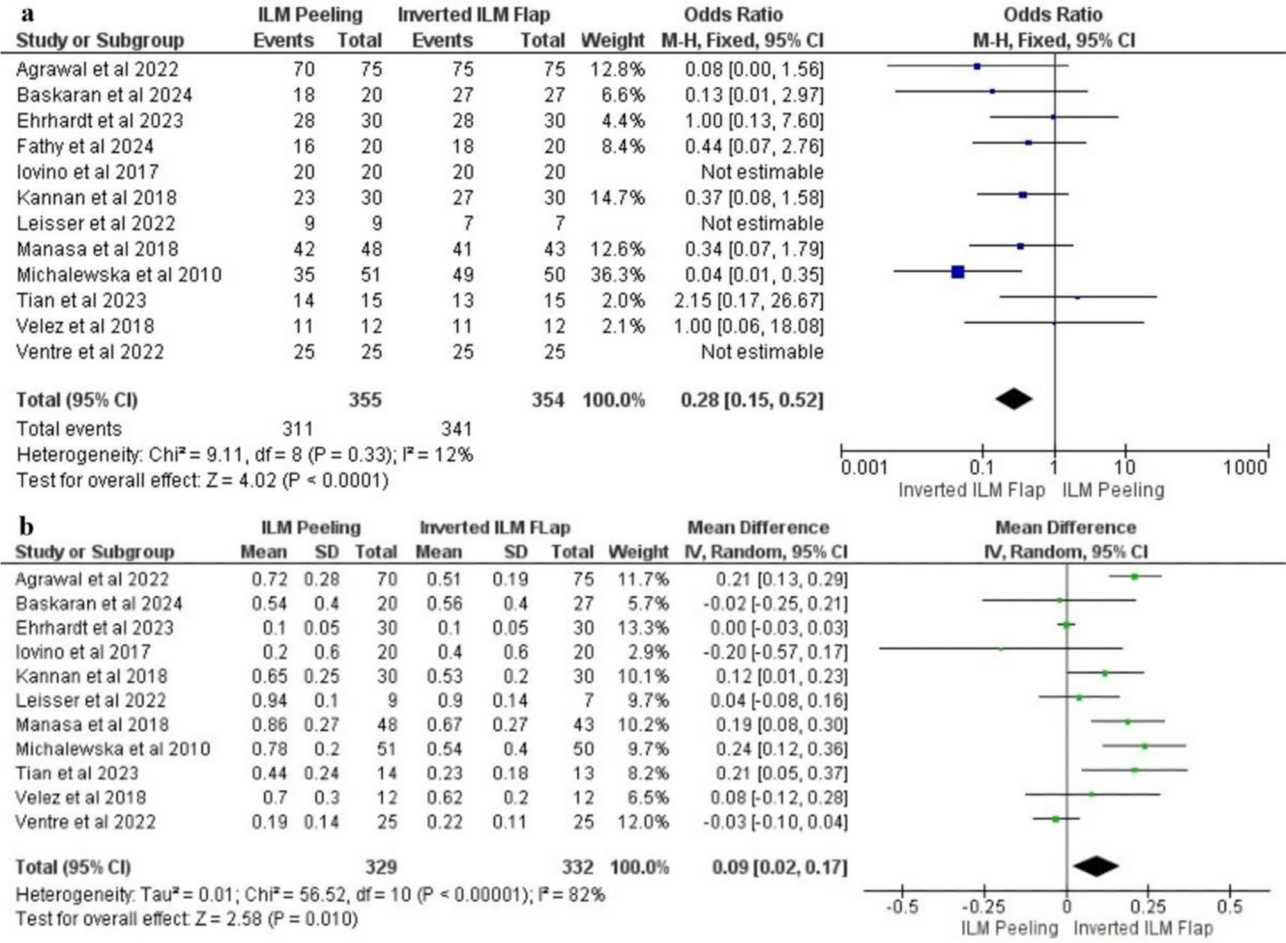
Forest plot of overall result. Anatomical closure (**a**) and visual outcomes post-procedure (**b**)

In the twelve included studies, anatomical closure was achieved in 311 out of 355 eyes with ILM peeling and in 341 out of 354 eyes with the inverted procedure. The fixed-effect model, which was used to pool the data (I^2^ = 12%, *P* < 0.33), and the inverted ILM flap procedure yielded significant results in terms of anatomical closure compared with ILM peeling [OR 0.28; 95% CI (0.15–0.52); *p* < 0.0001] Fig. 3a.

### Overall visual outcomes post-procedure

The pooled analysis of eleven RCTs at the final follow-up revealed high heterogeneity (*P* < 0.00001, I^2^ = 82%) and demonstrated significant improvement in postoperative VA, favoring the inverted ILM flap over ILM peeling [MD 0.09; 95% CI (0.02–0.17); *p* = 0.01] Fig. 3b.

### Visual outcomes at the 3-, 6-, and 12-month follow-ups

**Fig. 4:**
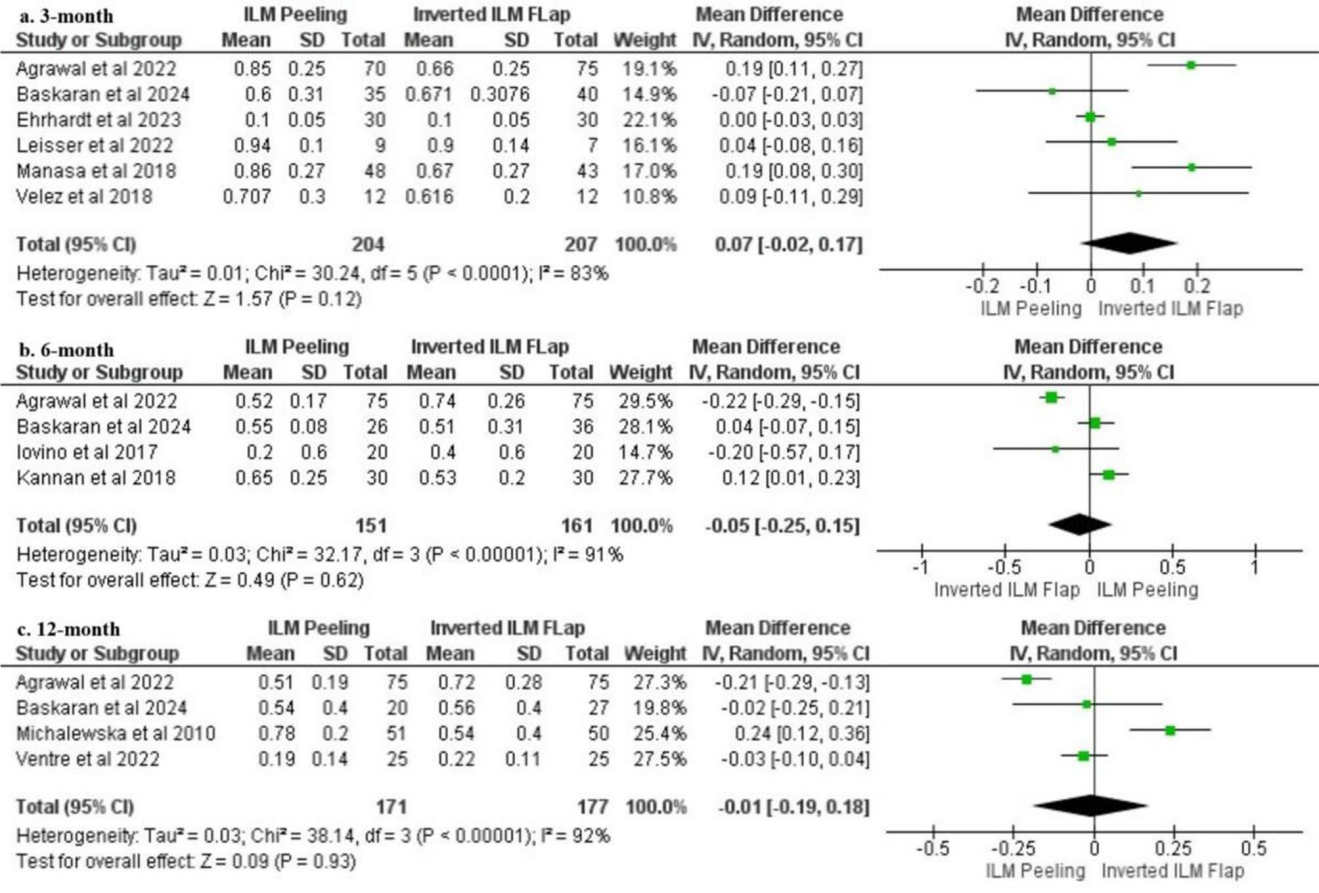
Forest plot visual outcomes based on follow-up. (**a**) 3-month (**b**) 6-month (**c**) 12-month follow-up

VA outcomes were analysed based on follow-up duration at 3, 6, and 12 months post-procedure. At 3 months, the data from six studies [14– [17, 19, 20] had an MD of 0.07; (95% CI -0.02-0.17; *p* = 0.12). At 6 months, four studies [12, 13, 17, 20] reported an MD of -0.05; (95% CI -0.25-0.15; *p* = 0.62). At 12 months, VA outcomes from four studies [5, 17, 18, 20] were MD -0.01; (95% CI -0.19-0.18; *p* = 0.93). These results indicate no statistically significant differences in postoperative VA between the ILM peeling and inverted ILM flap techniques across all follow-up durations Fig. 4.

### Macular closure at 3-, 6-, and 12-month follow-ups

**Fig. 5:**
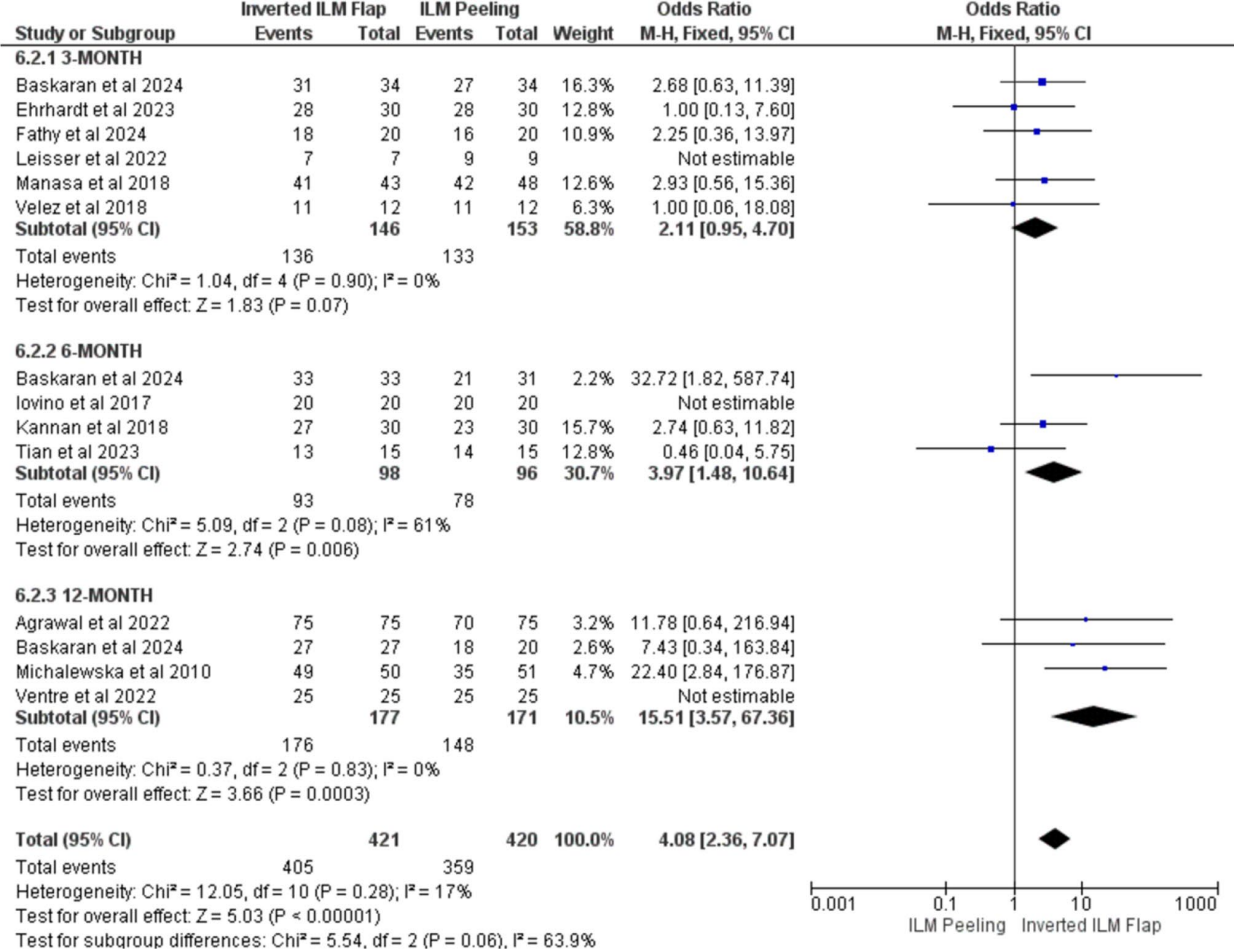
Forest plot macular closure based on follow-up time

Regarding follow-up duration, there was a trend of increasing odds of MH closure over time with the inverted ILM flap compared to the ILM peeling. Six studies reported data at the 3-month follow-up [14– [16, 19, 20, 22]. The OR was 2.11 (95% CI: 0.95–4.70), *p* = 0.07, indicating a positive trend, although it was not statistically significant. At 6 months, four studies were included [12, 13, 20, 21]. The OR increased to 3.97 (95% CI: 1.48–10.64); *p* = 0.006, indicating better results with an inverted flap. At the 12-month follow-up, the difference was highly significant, with an OR of 15.51 (95% CI: 3.57–67.36); *p* = 0.0003 for four studies [5, 17, 18, 20] strongly favoring the inverted ILM flap method for anatomical closure. These results suggest that the benefit of the inverted ILM flap technique becomes more apparent over time, particularly at the 12-month follow-up Fig. 5.

### Analyses of anatomical closure based on the type of closure

**Fig. 6:**
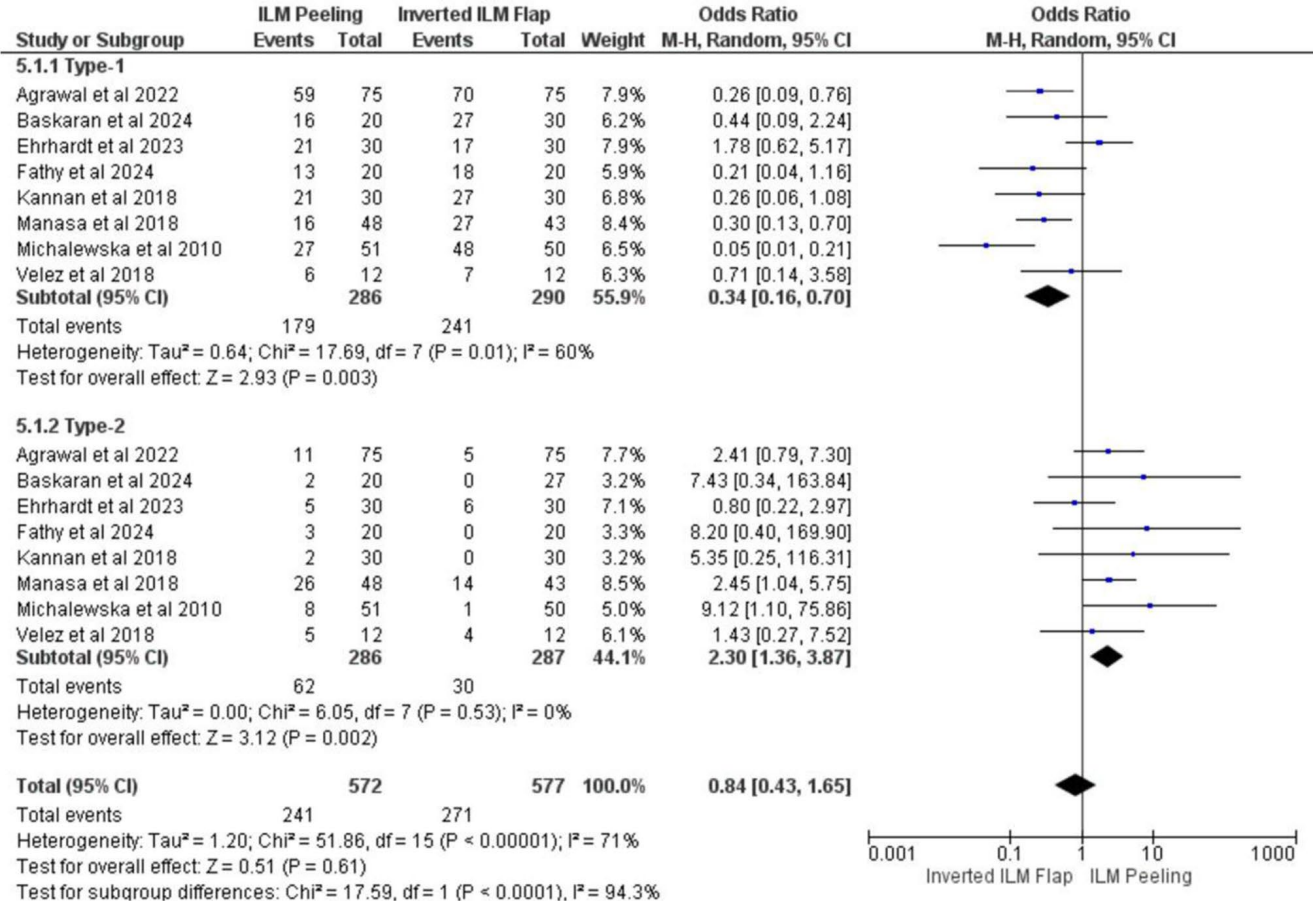
Forest plot anatomical closure divided by type of closure

Anatomical closure is divided into type 1 and type 2 [5]^13^– [17, 20, 22]. Type 1 closure is defined as complete closure of the macular hole with restoration of the normal retinal structure across all layers, while Type 2 closure refers to closure without full retinal layer reconstruction, where the retinal pigment epithelium is no longer exposed to the vitreous cavity, but the foveal contour remains disrupted [23]. In type-1 patients, 179 out of 286 eyes underwent peeling, and 241 out of 290 eyes underwent an inverted flap. In the type-1 closure subgroup, the OR was 0.34 (95% CI: 0.16–0.70); *p* = 0.003. The analysis revealed a significant association between the inverted flap and a greater degree of type-1 MH closure. In the group with type-2 MH closure, 62 out of 286 eyes had peeling and 30 out of 287 eyes had the inverted flap group, with an OR of 2.30 (95% CI: 1.36–3.87); *p* = 0.002, indicating that the ILM peeling procedure significantly tends to result in type-2 closure. The test for subgroup differences revealed statistically significant differences (p = < 0.0001), indicating that different techniques yielded different types of closure. The overall pooled analysis, including all closure types (Type-1 and Type-2), yielded an OR of 0.84 (95% CI: 0.43–1.65); *p* = 0.61, suggesting that there was no statistically significant difference in the overall macular closure rate between the two surgical techniques Fig. 6.

### Sensitivity analysis

In this analysis, outcomes were stratified by MH size (< 400 μm and ≥ 400 μm) to assess the impact of surgical techniques on VA and anatomical closure. For MH < 400 μm, the improvement in postoperative VA [15, 18, 19, 21] between peeling and inverted flaps was not statistically significant, MD 0.02 (95% CI: -0.04-0.09); *p* = 0.49, and the anatomical closure [15, 21] were not significantly different OR 1.37; 95% CI: 0.29–6.48; *p* = 0.69. In contrast, for MHs ≥ 400 μm, the inverted flap demonstrated a significant advantage in both visual improvement [5]^12^– [14, 16, 17, 20] (MD 0.15; 95% CI: 0.08–0.22; *p* < 0.0001) and closure rates [5, 13, 14, 16, 17, 20, 22] (OR 0.20; 95% CI: 0.10–0.42; *p* < 0.0001). The test for subgroup differences revealed statistically significant differences for VA (*p* = 0.006) and anatomical closure (*p* = 0.03), highlighting the superior effectiveness of the inverted flap method, particularly for larger MHs. Overall heterogeneity in postoperative VA remained high (I^2^ = 82%), and a marked reduction was observed in the ≥ 400 μm subgroup (I^2^ = 41%), suggesting greater consistency of results across studies in this group than in the < 400 μm subgroup, where heterogeneity remained high. The figure can be seen in the additional file, Fig. 1 for anatomical closure and Fig. 2 for VA.

### Publication bias

Publication bias was assessed via a funnel plot, which appeared symmetrical, indicating no significant evidence of publication bias among the included studies, see additional file Fig. 3.

## Discussion

The expected outcome improvement is increasing with the advancement of knowledge and therapeutic options for MHs. The inverted flap is a practical choice among the many therapeutic options for MH. In this meta-analysis study, from 12 RCTs, the primary outcome of anatomical closure was significantly greater than that of ILM peeling, yielding perfect closure rates of 80–95% in the cases. The development of techniques in the management of MHs is increasing and evolving, resulting in nearly 100% closure rates [24].

Similar to previous meta-analysis findings [25–31], this study also revealed that overall anatomical closure is significantly better with the inverted flap technique than conventional peeling (*p* < 0.0001). When analysed based on follow-up duration, the effectiveness of the inverted flap technique appears to increase over time, suggesting that it may yield better outcomes in long-term follow-up. The forest plot results indicate a pattern where longer follow-up durations are associated with more favorable results for the inverted flap technique. This trend is likely due to the gradual process of MH closure. In the study by Shiode et al., the ILM was described as a structural scaffold that supports the proliferation and migration of Müller glial cells. Once activated, these cells may release beneficial substances, such as neurotrophic factors and basic fibroblast growth factor (bFGF), which are also believed to be present on the ILM surface. When the ILM was placed on top of the MH, it can serve as a localised source of these growth-promoting molecules, potentially enhancing the healing process. These findings suggest that Müller cell gliolysis is stimulated by the placement of an inverted ILM flap, and the release of soluble factors like neurotrophic substances and bFGF plays a key role in MH closure. Previous studies showed that Müller cell gliolysis and migration are more effectively initiated in a physical scaffold such as the ILM [32].

Anatomical closure was further analysed based on closure types, categorised into type 1 and type 2. The analysis revealed that the inverted flap tends to result in type 1, whereas conventional peeling is more often associated with type 2 closure. It is hypothesised that type 1 closure leads to better visual outcomes associated with improved photoreceptor alignment and more complete retinal structural restoration [5].

Although the inverted flap technique improved overall visual outcomes (*p* = 0.01), further analysis was conducted to evaluate VA specifically at the final follow-up points reported in the studies at 3, 6, and 12 months. This analysis did not reveal statistically significant differences in visual outcomes at these individual time points. Improvements in VA largely depend on restoring the external limiting membrane (ELM) and the ellipsoid zone (EZ), which are critical components of photoreceptor microstructure and key prognostic factors for visual outcomes after MH surgery [33]. While effective for anatomical closure, the inverted ILM flap may mechanically interfere with photoreceptors at the edges of the MH. This interference can lead to abnormalities in the ELM and EZ within the foveal region, thereby hindering the recovery of foveal architecture and resulting in suboptimal visual outcomes postoperatively [34, 35]. In the first month following surgery, the inverted flap significantly improved VA more than peeling. However, the difference in VA between the two techniques diminished from the third month onward, with no statistically significant difference observed [31, 36, 37].

Moreover, while Müller cell gliolysis plays a supportive role in MH closure, excessive proliferation of glial tissue may damage retinal nerve cells and negatively affect visual recovery [38]. On optical coherence tomography (OCT), excessive gliolysis appears as a hyperreflective foveal lesion due to glial overgrowth. While glial cells aid in healing, excessive proliferation may block photoreceptor migration, disrupting foveal reconstruction and IS/OS junction recovery, leading to poor visual outcomes [39–41]. Previous studies have reported similar findings, indicating that anatomical closure of the MH does not always correlate with improved postoperative VA [13, 20, 28, 31, 34, 35, 42, 43].

Additional factors may also contribute to less favorable outcomes after MH surgery, such as large hole size (greater than 500 μm), insufficient ILM peeling, preoperative VA, flat-edge morphology, inadequate postoperative positioning, suboptimal use of gas tamponade, delayed treatment, high myopia and a history of ocular trauma, which can further reduce the chances of successful recovery. Moreover, coexisting conditions such as uveitis, macular disorders such as age-related macular degeneration (AMD), postoperative cystoid macular edema, and retinal detachment have also been linked to poor visual and anatomical results [5, 44–46].

Earlier studies have shown that preoperative VA and the MH size are essential determinants of postoperative visual impairment [42, 47]. One of the most critical predictors of visual outcome is the length of time the MH has been present. The evidence suggests that the chances of achieving successful visual recovery are reduced by approximately 50% when the hole persists for more than four months. Performing surgery sooner can significantly enhance visual outcomes. The presence of AMD can also negatively affect surgical outcomes. In elderly patients without AMD, good VA can still be achieved following MH repair. Notably, age does not appear to impact the rate of MH closure directly [46, 48]. Large MHs are challenging due to the difficulty in achieving anatomical closure. As the diameter increases, the likelihood of successful closure decreases. A study by Steel et al. [46] reported failure rates as follows: 1.1% for < 400 μm, 3,1% for 400–599 μm, 10.5% for 500–599 μm, and 13.5% for ≥ 600 μm. Ch’ng et al. [49] observed increasing failure rates in large MHs, with 2% for 400–477 μm, 9% for 478–558 μm, 6% for 559–649 μm, and 24% for 650–1416 μm. Michalewska et al. were the first to apply the inverted flap for treating MHs with diameters greater than 400 μm, reporting highly favorable outcomes [5]. Subsequent studies confirmed these findings, consistently demonstrating improved anatomical closure in MHs exceeding 400 μm in diameter [28, 42, 47]^50^– [52].

Although no significant post-vitrectomy complications were reported in the included studies, long-term outcome data were not provided, vitrectomy is known to be associated with several potential complications. One common complication is epiretinal membrane formation, which may require reoperation. The inverted ILM flap may act as a scaffold for Müller cell proliferation, and residual vitreous cells and collagen can promote epiretinal membrane development. Trapped cells between ILM layers during flap placement have also been linked to increased epiretinal formation [53]. Cataract progression is frequently observed post-vitrectomy, likely due to changes in intraocular oxygen levels and aqueous humour circulation. Other potential complications include elevated intraocular pressure, ILM flap dislocation, visual field defects, retinal detachment, vitreous hemorrahge, ocular hypotony, IOL dislocation, RPE changes and endophthalmitis [54].

This study has several limitations that should be acknowledged. First, although a meta-analysis was conducted using data from multiple RCTs, heterogeneity may have influenced the results. Second, VA outcomes across studies were measured at different follow-up times, and the number of studies analysed based on follow-up times remains limited, which may affect the robustness of time-based outcome comparisons. Third, not all included studies reported detailed information regarding MH duration, which is known to affect functional outcomes significantly. Fourth, a third closure pattern called “bridging flap closure”, in which the ILM flaps cover the hole without true retinal tissue apposition, was not clearly identified or classified separately in the included RCTs. Fifth, none of the included studies reported intraoperative or postoperative complications or provided sufficient data on cases that failed to achieve closure, thereby limiting our ability to assess the safety profile and failure rates of both techniques. Future studies are expected to provide more comprehensive and detailed data, enabling a thorough evaluation of the efficacy and safety of both surgical techniques.

## Conclusion

The inverted ILM flap technique demonstrates superior anatomical closure for MH management, particularly in cases with larger MH diameters. However, a statistically significant improvement in VA outcomes was not consistently observed, potentially influenced by factors such as retinal layer restoration and MH chronicity. Further investigations through larger-scale studies are warranted to establish the impact on functional outcomes.

## Electronic supplementary material

Below is the link to the electronic supplementary material.

Supplementary Material 1

## Data Availability

No datasets were generated or analysed during the current study.

## Abbreviations

ILM: Internal limiting membrane
MH: Macular hole
RCT: Randomized controlled trial
OR: Odds ratio
MD: Mean difference
CI: Confidence interval
VA: Visual acuity
BCVA: Best corrected visual acuity
RoB: Risk of bias tool
OCT: Optical coherence tomography
AMD: Age-related macular degeneration
IOL: Intraocular Lens
