# Supplementary material for: Comparison of ILM peeling vs. inverted ILM flap for macular hole closure and visual outcomes: systematic review and meta-analysis
Source: Int J Retina Vitreous. 2025 Jul 17;11:81. doi: 10.1186/s40942-025-00707-z (PMC12273303; doi:10.1186/s40942-025-00707-z)
Supplement: Supplementary file 1 — Supplementary Material 1 [file 40942_2025_707_MOESM1_ESM.zip › 40942_2025_707_MOESM1_ESM/40942_2025_707_MOESM1_ESM.pdf]

Search Strategy

**PubMed (NCBI)**		
#1	(macular hole) OR (macular holes)	8.839
#2	(inverted ilm flap) OR (inverted internal limiting membrane flap)	368
#3	(((ilm peeling) OR (internal limiting membrane peeling)) OR (conventional ilm peeling)) OR (conventional peeling)	2.588
#4	#1 AND #2 AND #3	167

**ScienceDirect**	
inverted ilm flap and ilm peeling and macular hole	109

**Cochrane Library & ClinicalTrials.gov**		
#1	MEsh DESCRIPTOR: [Retinal Perforations]	298
#2	(macular NEXT (hole* or holes*)):ti,ab,kw	532
#3	#1 OR #2	606
#4	(ilm peeling or *conventional ilm peeling*):ti,ab,kw	216
#5	(inverted ilm flap NEXT (technique* or method* or procedure*)):ti,ab,kw	34
#6	#3 AND #4 AND #5	23<br>Cochrane Library = 1<br>ClinicalTrials.gov = 22
