# Supplementary figures and images for: Comparison of ILM peeling vs. inverted ILM flap for macular hole closure and visual outcomes: systematic review and meta-analysis
Source: Int J Retina Vitreous. 2025 Jul 17;11:81. doi: 10.1186/s40942-025-00707-z (PMC12273303; doi:10.1186/s40942-025-00707-z)

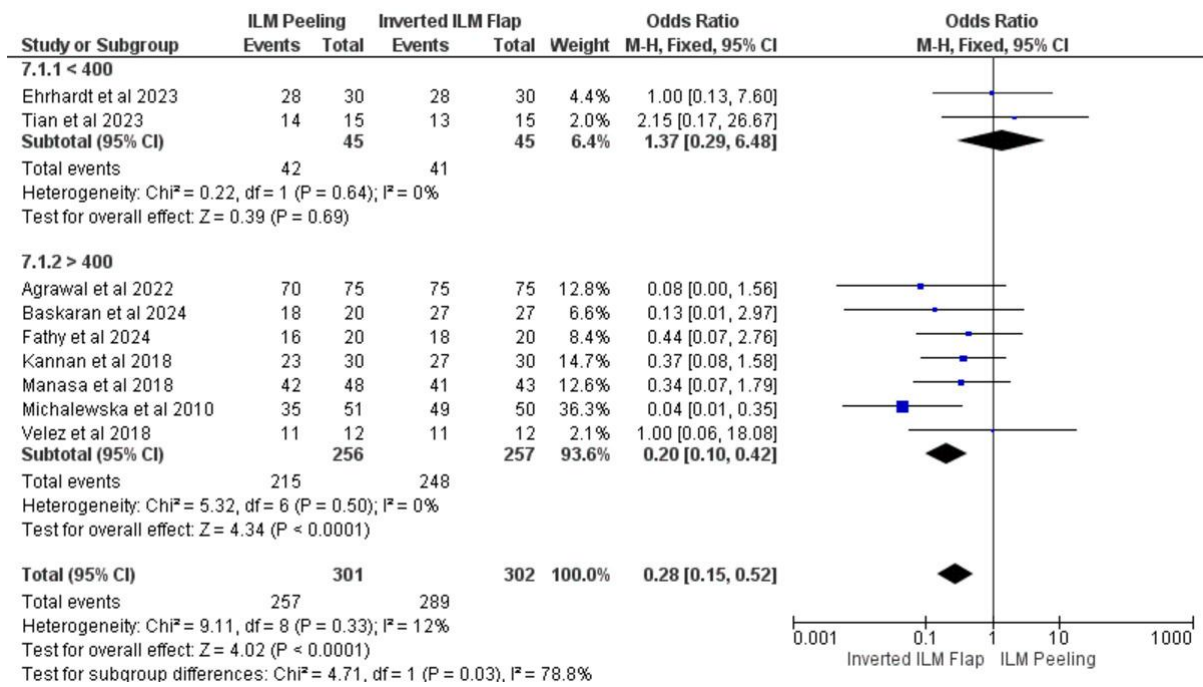

**Add. Fig. 1 Forest plot of anatomical closure by size of macular hole**

Supplement: Supplementary file 1 — Supplementary Material 1 [file 40942_2025_707_MOESM1_ESM.zip › 40942_2025_707_MOESM1_ESM/40942_2025_707_MOESM3_ESM.pdf]

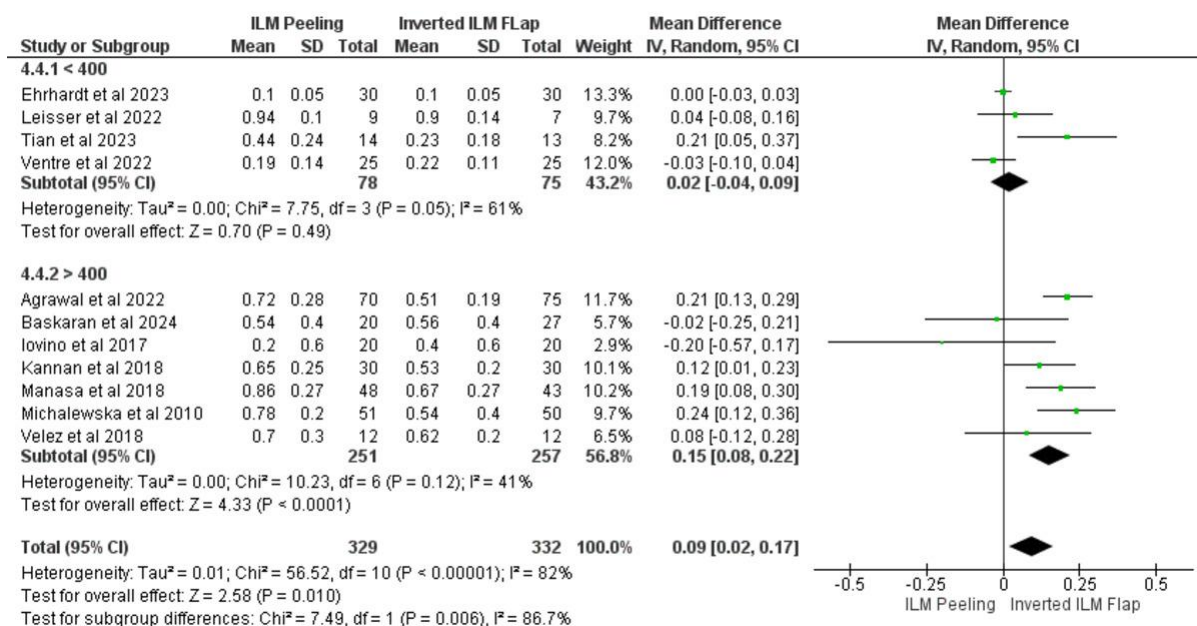

**Add. Fig. 2 Forest plot of visual outcomes by size of macular hole**

Supplement: Supplementary file 1 — Supplementary Material 1 [file 40942_2025_707_MOESM1_ESM.zip › 40942_2025_707_MOESM1_ESM/40942_2025_707_MOESM4_ESM.pdf]

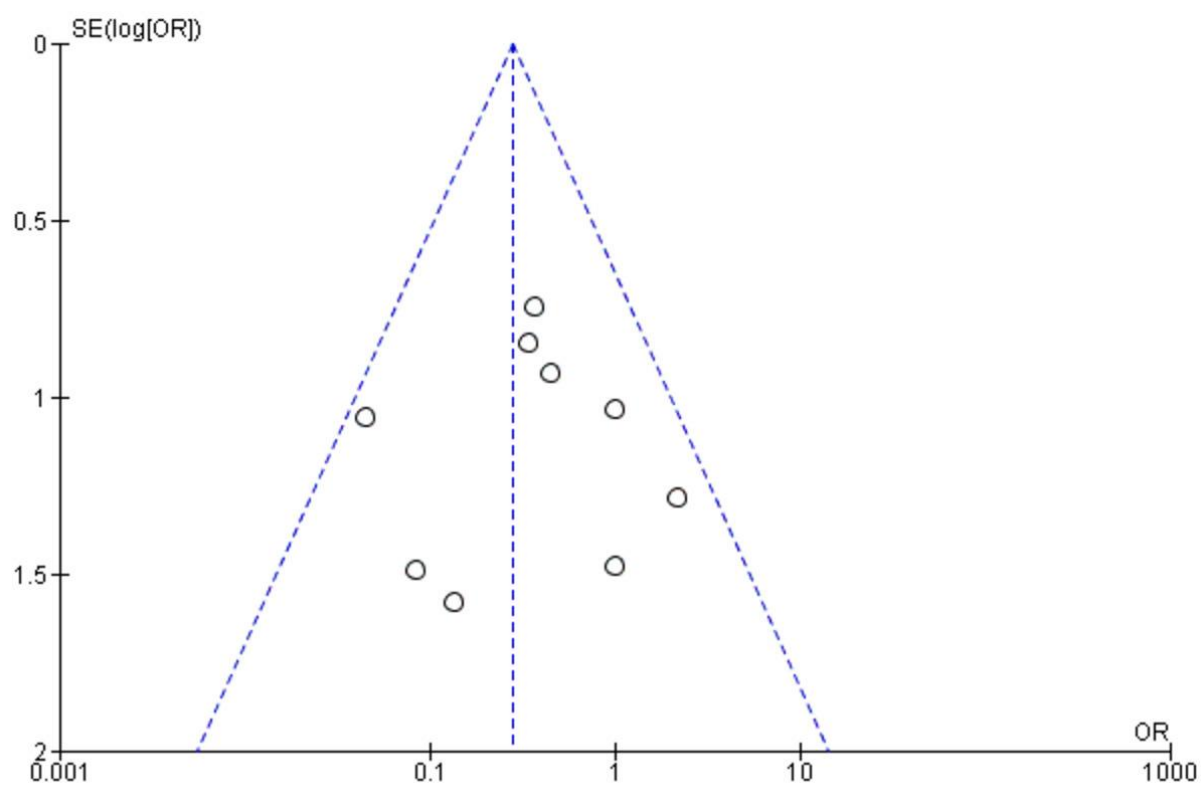

**Add. Fig. 3** Funnel plot of primary outcome: anatomical closure.

Supplement: Supplementary file 1 — Supplementary Material 1 [file 40942_2025_707_MOESM1_ESM.zip › 40942_2025_707_MOESM1_ESM/40942_2025_707_MOESM5_ESM.pdf]
